# Lessons From the February 2023 Turkish Earthquake

**DOI:** 10.7759/cureus.71042

**Published:** 2024-10-07

**Authors:** Elsy Jabbour, Yara Mouawad, Danielle Abou Khater, Mariana Helou

**Affiliations:** 1 Emergency, Lebanese American University School of Medicine, Beirut, LBN

**Keywords:** disaster preparedness, disaster response, earthquake, natural disaster, turkey

## Abstract

On February 6, 2023, Turkey was struck by the most powerful earthquake recorded since 1939, leaving millions of people devastated and homeless with over 36,000 casualties. According to the Ministry of Health, at least 50% of the major health centers were damaged, with only about 30% of the pre-existing doctors able to help. The earthquake occurred during a harsh winter and amidst an ongoing humanitarian complex situation in Syria resulting from the Syrian civil war that started in 2011. This report aims to present the Turkish 2023 earthquake preparedness and mitigation efforts. The objective of this article is to extract valuable insights and identify measures that can be taken to improve response based on the lessons learned from this event.

## Editorial

Introduction

On February 6, 2023, two consecutive earthquakes, measuring 7.8 and 7.5 magnitudes on the Richter scale, struck northern and western Syria and southern and central Turkey. The epicenter of the earthquakes was located near Gaziantep, a city in southeastern Turkey. The disaster resulted in over 36,000 casualties and left millions of people without homes in both countries [1]. Thousands of buildings collapsed, including hospitals and schools. According to the Ministry of Health, at least 50% of the major health centers were damaged, with only about 30% of the pre-existing doctors able to help. Additionally, more than 2000 aftershocks followed the initial earthquakes, as of March 1. The earthquake occurred during a harsh winter and amidst an ongoing humanitarian complex situation in Syria resulting from the Syrian civil war that started in 2011 [2].

This report aims to present the Turkish 2023 earthquake preparedness and mitigation efforts. The objective of this article is to extract valuable insights and identify measures that can be taken to improve response based on the lessons learned from this event.

Human impact

The medical conditions observed among the survivors were predominantly physical in nature, ranging from fractures to crush injuries and infectious complications later on. Additionally, many individuals suffered from hypothermia and frostbite due to severe weather conditions. There were also cases of respiratory distress resulting from smoke and dust inhalation injuries. Patients who were homeless or lacked access to necessary medications, including those with chronic conditions such as cancer, diabetes, and hypertension, were particularly vulnerable [3].

According to a review of 34 studies, the epidural hematoma was found to be the most frequently occurring type of intracranial bleeding associated with earthquakes. Head trauma was the primary cause of fatal injuries and the third most prevalent injury type, accounting for 16.6% of cases, following lower extremity injuries (36.2%) and upper extremity injuries (19.9%) [4]. The true number of the types of injuries from this disaster remains unknown till the date of this report.

International response

During a televised speech, President Recep Tayyip Erdogan announced that over 70 countries had offered assistance and stated that the affected regions would be under a level four emergency state for three months [5].

Following the earthquake, both Syria and Turkey made appeals for international aid. The Red Cross promptly responded by sending blood products, food, and supplies for those who were left homeless. In addition, the World Health Organization and numerous other organizations sent medical equipment and food to both countries. Emergency response teams, specially trained in disaster situations, were also dispatched to assist with the retrieval of individuals from the rubble and provide initial medical aid. The shortage of trauma kits, serum, and painkillers became apparent in hospitals, and heavy machinery was urgently required to clear the rubble [6].

Lessons learned

The Health Emergency and Disaster Risk Management (EDRM) Framework highlights the significance of the four stages of a disaster plan: mitigation, preparedness, response, and recovery [7]. Understanding a disaster plan is critical to saving lives. During such disasters, the response efforts can be categorized into three primary areas: providing initial medical assistance and transporting individuals to healthcare facilities, supplying food and medical resources, and providing shelter for those who are homeless.

To ensure that these needs are addressed in a timely manner, it is necessary to have disaster plans in place. For example, the potential locations for temporary medical centers should be identified and evaluated based on the areas most affected and the extent of damage to the roads. Transportation of victims during disasters is hindered by the damage to infrastructure, despite the fact that an ambulance has the capacity to carry up to ten individuals, one of whom may be severely injured [8]. Numerous studies have noted that triage is the most challenging yet crucial aspect of medical care during disasters. Communication difficulties and the perception of each victim's condition are critical addition to the complexity of triage. Effective triage is essential for saving as many lives as possible [9].

A framework for the distribution of temporary hospitals in the event of a disaster was proposed in a case study conducted in Istanbul. According to the study, a physician was assumed to have the capacity to simultaneously attend to nine mild cases and three major cases [4].

Effective communication plays a crucial role in managing a disaster. With the emergence of social media, sharing information has become easier, which can be useful for non-medical volunteers and survivors in the early response phase. However, ensuring the safety of the treating team and the victims should always be prioritized, especially when working in out-of-hospital settings, as in any other mass casualty [10].

As the world continues to grapple with the ongoing COVID-19 pandemic, the occurrence of a natural disaster such as an earthquake could exacerbate the risk of spreading various respiratory viruses. In Syria, where millions of vulnerable individuals are already living in unsanitary conditions, there is a heightened risk of a cholera outbreak, which the country has been struggling to contain for several months. Furthermore, individuals who are left without shelter may face additional problems such as dehydration and infections. Tetanus is also a significant concern, as access to immunoglobulin may be limited due to increased demand and scarce resources. The Syrian population is particularly susceptible to tetanus due to inadequate immunization, which has been compounded by the civil war and economic crisis. Turkey is known to have endemic Leishmaniasis, and the overcrowding caused by this disaster could potentially lead to an increase in its transmission [5]. The risk of disease growth can be avoided by establishing an infectious surveillance system that will detect and track the outbreak, promote hygiene awareness, and prevent overcrowding in the shelters.

Using low-quality building structures also played a major role in increasing the damage. Experts raised the issue of illegal buildings and old structures not matching the standards. Thus, this event highlights the importance of adherence to proper construction standards, and entrusting this responsibility to well-trained engineers. Turkey and Syria are seismic-prone regions and unfortunately, this disaster will not be the last. Therefore, building codes are key to ensuring earthquake-resistant buildings.

Table 1 summarizes the disaster-related elements evaluating the challenges following the drastic damage and many actions that can be taken to improve the readiness to face similar future situations [4,11].

**Table 1:** Challenges and actions to be taken.

Challenges	Actions to be taken
Preparedness	Training and simulation exercises in the hospitals. Implementation of a disaster plan, emergency policies and protocols. Setting up potential temporary healthcare facilities to relieve overcrowded facilities. Assigning areas of gatherings. Optimizing facilities’ capacity. Awareness campaigns on first aid, and self-protection.
Rapid response	Proper triage and patients distribution. In-hospital organization and communication between different services (emergency department, radiology, operation room…).
International communication	Coordination with national and international organizations. Management of donations. Providing updates on the real-time situation.
Psychological support	Psychological Debriefing to prevent the development of post-traumatic stress disorder and other negative consequences. Providing psychological sessions for the victims, the responders, and the affected population.
Security and safety	Decontamination, evacuation, and safe transport of victims. Ensuring crowd control. Offering shelter for victims along with basic life needs such as food and sanitary equipment. Conducting hazard analysis and security studies on the ground for potential threats. Communication with the security officers. Establishing a disease surveillance system. Tools and awareness campaigns to help citizens to verify the safety of their structures.

Conclusion

Disaster operations are key components to mitigate the adverse effects of the damage, especially in resource-limited countries. The real killer was once again not the seismic event, but the vulnerable structures in both countries and the poor preparation. During this disaster, two main components aggravated the impact of the injuries, the bad structure of the buildings in both countries and the lack of coordination with the absence of enough manpower and equipment. This earthquake reminds us about the importance of conducting realistic studies to make the optimal benefit of available resources.
